# Psychological assessment in forensic settings: new methodological conceptions and ethical considerations

**DOI:** 10.3389/fpsyg.2026.1825338

**Published:** 2026-05-29

**Authors:** Alfredo Verde, Emanuela Saveria Gritti

**Affiliations:** Università di Genova, Dipartimento di Scienze della Salute - Sezione di Scienze Medico-forensi, Genova, Italy

**Keywords:** abductive reasoning, case formulation, cognitive bias, dual-process theory, forensic psychological assessment, multimethod assessment, nomothetic–idiographic integration, Peirce

## Abstract

Forensic psychological and psychiatric evaluations—understood here as assessments addressing legally operative questions such as, for instance, competency to stand trial, criminal responsibility (sanity), violence risk, but also those on legal capacity and child custody—must meet stringent standards of scientific rigor, transparency, and replicability, yet the reasoning processes underlying clinical formulation require further theoretical articulation. This paper operates at an explicitly metatheoretical level: it does not propose a new clinical procedure, but an epistemological framework grounded in C. S. Peirce's theory of abduction. Abduction designates a third form of inference, irreducible to both induction and deduction: whereas deduction derives a necessary conclusion from a rule, and induction generalizes a rule from observed cases, abduction moves from a surprising fact to the most plausible explanatory hypothesis—a provisional conjecture that requires deductive and inductive testing. Within this framework, forensic case formulation is reconceptualized as an iterative, multi-level abductive process in which idiographic formulations must be systematically anchored to nomothetic data, safeguarded by dual-process reasoning and multimethod and Process-Focused assessment against distortions such as forensic confirmation bias, first-impression effects, and malingering. A novel theoretical contribution is a fractal model of abductive reasoning: clinical inference replicates its structure from micro-abductions on individual signs, through meso-abductions on diagnostic patterns, to macro-abductive formulations. Diagnostic errors can be understood as a “collapse of the fractal.” Three vignettes from Italian proceedings illustrate the framework. The paper concludes with methodological and ethical recommendations, arguing that diagnostic error in forensic contexts carries both epistemic and ethical consequences.

## 1 Introductory remarks

This paper examines why the scientific status of psychological and psychiatric evidence has been questioned, differentiates among the levels of scientific rigor achievable in expert evaluations, and formulates recommendations for practitioners.

Drawing on Peirce's dual formulation of abduction and on the continuum of abductive forms, the paper reconceptualizes forensic psychological assessment as an iterative, multi-level abductive process in which idiographic clinical formulations must be systematically anchored to nomothetic psychodiagnostic data. This integration is illuminated through the concept of forensic case formulation as an abductive enterprise, through dual-process models of clinical reasoning, and through multimethod and Process-Focused assessment, which provides the primary methodological safeguard against the distortions introduced by unacknowledged micro-abductions—from forensic confirmation bias and first-impression effects to self-presentation strategies such as malingering. A novel theoretical contribution is a fractal model of abductive reasoning: clinical inference replicates its logical structure at progressively higher scales, from micro-abductions on individual signs and test scores, through meso-abductions on diagnostic patterns, to macro-abductive case formulations. Diagnostic errors—whether produced by cognitive biases, narrative distortions, or deliberate manipulation of assessment data—can be understood as a “collapse of the fractal”, a short-circuiting of intermediate verification steps.

The framework is designed to be general in scope: different legal systems configure the relationship between clinical assessment and legal decision-making in different ways—what North American forensic psychology designates as a “forensic evaluation” often corresponds, in civil law systems such as the Italian one, to a broader mandate that nevertheless requires comprehensive clinical formulation. For instance, the assessment of “*capacità di intendere e di volere*” (criminal responsibility), in the Italian criminal law, formally constitutes a forensic evaluation—yet it requires a clinical evaluation of the examinee's personality structure, psychopathological history, and contextual factors. The terminological boundary is itself legally and culturally variable. More fundamentally, the abductive framework we propose operates at the level of the diagnostic reasoning process, which is structurally invariant across legal systems. What varies is the final abductive step: the translation of clinical constructs into the legally operative categories of the relevant jurisdiction. This translation is itself an abductive operation, and its recognition as such is one of the practical contributions of our framework. Three forensic clinical vignettes from Italian proceedings illustrate how failures in diagnostic reasoning can occur in practice and how rigorous abductive reasoning can integrate heterogeneous data into a defensible formulation. The paper concludes with methodological recommendations and ethical considerations, arguing that diagnostic error in forensic contexts carries not only epistemic but also ethical consequences.

## 2 The scientific status of psychological diagnoses

The problem of the scientific status of evidence in courts has long been debated, since it concerns the possibility of using research results at the public level. Regarding forensic sciences, numerous criteria have been formulated to enable the validation of findings from various disciplines in legal proceedings. From this standpoint, as is well known, common law courts—particularly those of the United States—have been able to formulate general principles, from the Frye standard to the Daubert criteria, which require the possibility of empirically testing theories, the necessity of peer review and publication of results, the formal assessment of the possible rate of erroneous results, and the acceptance of the investigative methodology by the broader scholarly community (Goodman-Delahunty, 1997).

The scientific status of psychological and psychiatric diagnoses depends, obviously, on the scientific quality of the description of the patient's condition and on the generalizability of the conclusions reached; on the one hand, one must consider the value of clinical hypotheses developed through interviews, and on the other, one must assess the scientific improvement achieved through the use of psychological testing instruments. To put it briefly, simple clinical interviews do not allow sufficient reliability to be achieved across different examiners, to the extent that it is now accepted that, given these limitations, it is necessary to resort to a battery of testing tools that enable the achievement of sufficient inter-rater reliability, obviously considering and assessing their empirical validity (Heilbrun, 1992); but also the scientific level of psychological testing began to be thoroughly assessed (DeMatteo et al., 2020; Mihura et al., 2013; Neal et al., 2019).

To better understand such an issue, one has to revisit the foundational principles of diagnosis: the initial stage consists of the clinician's preliminary impression, which is subsequently refined through structured evaluations that align observed traits or symptoms with a nosographic framework to delineate precise configurations. Such procedures can be repeated, and each being confirmed or refuted when confronted with the case material. What comes out of the process is a description of a particular symptom or personality trait, or of a cluster of traits, that defines the presence of a disorder or of a characteristic of the personality. The problem is that each assessor may observe different aspects, and the diagnoses may not coincide. In other words, the inter-rater reliability of the diagnosis is low, and many efforts have been made to improve it: not only broadly, through successive revisions of DSM diagnostic criteria (Aboraya et al., 2006) aimed at enhancing interrater reliability, but also within the specific domain of psychoforensic assessment, which is our concern here (Grisso, 2003; Mossman and Somoza, 1991). As we shall see, “scientificness” can be a flag around which researchers rally to validate their practices and is considered by scholars a necessary level to legitimate their admissibility in courts. Put differently, the idiographic level should be confirmed by nomothetic data, and discrepancies should be carefully examined for the sake of a more thorough case formulation. It is precisely this insufficiency of purely nomothetic evidence in adversarial legal contexts—its inability to account for the idiographic singularity of the individual case without interpretive work—that motivates the epistemological turn the present paper proposes.

Until not many years ago, the diagnostic procedure was mainly conceptualized using the old concepts of Aristotelian logic (the so-called hypothetico-deductive model), referring to induction and deduction, which were theorized as the bases of clinical reasoning: induction would guide the formulation of a hypothesis, given certain characteristics of the symptoms, and deduction would allow one to empirically test whether other relevant facts could confirm or disconfirm it (Ward and Haig, 1997). But it is our opinion that the nomothetic level, in order to be applied to a singular individual, must give way to the idiographic, shaping their psychological portrait in its uniqueness.

To apply to such sophisticated reasoning only the logic of the Aristotelian-Galilean method (based on induction and deduction) is, according to the recent developments of scientific reasoning, too simplistic, and does not discriminate between the first level of what is called “induction” and the more sophisticated levels of such procedure.

Before proceeding, two clarifications are necessary. First, the contribution of this paper is metatheoretical rather than procedural: we do not claim that skilled forensic evaluators fail to reason abductively. On the contrary, the argument is that they do reason abductively—but without an explicit epistemological framework that names, transmits, and critiques this process. The analogy is instructive: physicians practiced what we now call germ theory before Koch articulated it. Articulation matters because it creates teachable standards, identifies failure modes, and opens practice to systematic critique and refinement. Second, the paper's scope is intentionally cross-jurisdictional in reason of the distinctions between country level legal systems that call assessors to differently translate clinical constructs into the legally operative categories of the relevant jurisdiction. The contribution of C.S. (Peirce 1931), in this regard, has been fundamental in formulating a “new old” model, based on the continuous, reiterative passage from the observation of data to theory and back: an inference through which the scholar perceives relationships between facts by picking out causal links and/or analogies, connecting a fact and a possible explanation of it. It is also defined in the literature as conjecture, retroduction, or hypothetical inference. This procedure enables explanations to be worked out; it does not, however, yield certain, necessary findings, but rather possible or more or less probable ones, to be verified. The definition is, however, complicated by the fact that, during the course of his production, Peirce adopted two different definitions of this stage of reasoning: the first defining a logical process of inference, the formulation of a hypothesis that is separate and distinct from those of induction and deduction, and the second is represented by the construction of a conjecture (called “abduction”), which would then be verified through the known inductive-deductive procedures.

Peirce's first definition of abductive inference was formulated within the Aristotelian framework of the syllogism, distinguishing it sharply from the other two forms of reasoning (Peirce, 1931, 2.623). In deduction, a general rule is applied to a particular case to yield a necessary result: the movement is from the universal to the particular, and the conclusion is logically compelled. Induction proceeds in the opposite direction: from the observation of a result in a specific instance, and the identification of the case as belonging to a class, one infers the general rule governing that class; the conclusion is probabilistic and revisable. Abduction—which Peirce at this stage terms “hypothesis”—inverts the deductive movement differently: given a rule and an observed result, one posits the unobserved case that would account for it. Unlike deduction, the conclusion is not necessary; unlike induction, the movement is not from particular instances to general laws, but from a surprising or anomalous fact to the most plausible explanatory conjecture. This is the third inferential form that constitutes the logical core of clinical diagnosis: the clinician moves from observed signs and symptoms (result) and theoretical knowledge about their typical configurations (rule) to a hypothesis about the underlying condition (case).

After 1891, Peirce inserted abductive inference into methodological processes: from this point of view, abduction becomes the formulation of a new hypothesis, and deduction and induction the ways in which this hypothesis can be verified.

We have seen that in contemporary discussions on the epistemology of clinical diagnosis, it is customary to invoke the Aristotelian–Galilean scientific method, as consolidated through Bacon's program, as if psychological assessment could be anchored to a purely deductive–inductive framework immune from interpretive uncertainty. Yet, as Hart et al. (2011) make clear, the actual logic that underlies clinical reasoning is abductive in the Peircean sense: the clinician identifies relationships among signs, selects causal connections or analogies, and formulates explanatory hypotheses capable of rendering the patient's condition intelligible. Such hypotheses do not carry the force of necessity; they represent plausible conjectures whose epistemic value depends on subsequent verification through deductive and inductive procedures. This is why Peirce's two formulations of abduction are operative in clinical practice. In his early, syllogistic formulation, abduction is the inferential move through which, given a rule and a result, one posits a case. In his later, methodological conception, abduction becomes the initial stage of scientific inquiry, the only moment in which genuinely new ideas emerge before deduction clarifies their implications and induction tests their empirical adequacy. When the clinician interprets behavioral patterns, symptom constellations, test data, and historical information and constructs an explanatory account, they are performing precisely this abductive act; and when they design further assessments to determine whether the predicted consequences follow, they are submitting this act to scientific scrutiny.

From within the theoretical distinctions articulated in the passage, scholars have traced a continuum of abductive forms that maps seamlessly onto clinical diagnostic practice. (Eco 1983) designates as “hypercodified” those abductions in which the relevant rule is fully established and uniquely applicable, so that the inference seems almost deductive. (Bonfantini and Proni 1983), on the other hand, underscore how, in such cases, the apparent logical strength of the conclusion may mask its abductive and therefore fallible nature. But Eco has also described another, less certain, form of abduction, calling it “hypocodified”, which can apply, as Magnani (2001) and other scholars (Rapezzi et al., 2005; Smargiassi et al., 2017) have asserted, to diagnosis in medicine, defining it as “selective” abduction, where multiple rules can plausibly account for the observed fact and the clinician must determine which explanatory model best fits the case. The probabilistic nature of the diagnosis, and the employment on hypothetical reason in the assessment process, has been underlined also by authors who contributed to semeiotics in medicine (Scandellari, 2005).

At the opposite end of the spectrum lies “creative” abduction, as described by (Hanson 1958) and in Peirce's mature work, where one introduces a new explanatory rule to make sense of a complex or anomalous presentation. Clinicians resort to this form of abduction in—the not so rare—cases that resist existing diagnostic categories, where symptomatic complexity, overlapping syndromes, or discordant test results require the construction of a personalized explanatory framework. Such formulations, however, should be tied to other, less creative and more observative abductions, and should also fit, in particular when a forensic formulation is needed, with the available nomothetic material.

This continuum, in which the conclusion of one abductive step becomes the premise for the next, is the essence of clinical formulation: an initial hypothesis about symptomatic organization becomes the basis for inferences about underlying mechanisms, which in turn inform expectations about contextual sensitivity, interpersonal functioning, risk, or future behavior. Over the course of clinical observation, new information either confirms, nuances, or contradicts earlier inferences. The final formulation is thus a stratified narrative composed of multiple interconnected abductive moves. It is “narrative” not in a literary sense, but in the epistemological manner described by (Eco 1983) and by (Bruner 1991). (Oatley 1996) emphasizes that narrative structuring is not alien to scientific explanation, since it is one of the principal modes through which human cognition organizes and understands experience. In this direction, Kenneth Burke's dramatistic categories—act, scene, agent, agency, purpose— (Burke, 1945) can clarify the content of a complete clinical formulation: what is occurring, in what context, who the patient is as an agent endowed with dispositions and vulnerabilities, through what mechanisms processes unfold, and toward which ends symptoms may be directed. Poetically, also Carlo Ginzburg's reflection on the hunter reading traces provides an apt metaphor (Ginzburg, 1986): the clinician reconstructs a life and a mind based on scattered, often ambiguous signs.

The passage also emphasizes an essential caution: the further abductive reasoning moves away from observable data and from inductively grounded probabilities, the greater the risk of error.

## 3 The abductive integration of clinical observations and test material in psychoforensic evaluations

If the application of abduction theory to medical diagnoses is easier, given that “hard” data (i.e., measures of substances in blood, images of the internal of the body, etc.) is easily available (Stanley and Campos, 2013), psychological diagnosis is much more difficult, since it investigates the characteristics of the psyche, which is a metaphorical construct, and initial diagnostic abductions do not depend on observation of physical signs as in medicine. The temptation to infer personality structure, motivational dynamics, or undocumented history from limited symptomatic material can lead to narratives that appear coherent but are not empirically grounded. For instance, in a case where an individual is being evaluated for an alleged violent crime against their partner that resulted in homicide, it would be flawed—if not outright tendentious— to infer a severe obsessive-compulsive disorder from the defendant's “perfect cleaning” of the crime scene, as such meticulous behavior might also be consistent with psychopathic features, reflecting a deliberate, perfectionistic attempt to conceal the crime. Even more problematic, in terms of diagnostic reliability and the assessment of mental sanity, would be to use such a flawed diagnostic conclusion regarding a presumed OCD as a mitigating circumstance for the crime committed. In this context, an in-depth assessment of the case, taking into account both personality test results and collateral information on the defendant, becomes crucial. In other words, the best safeguard lies in continuously considering alternative explanations, weighting them according to the number and strength of supporting premises, and seeking empirical confirmation whenever abductive reasoning ventures far from the available evidence.

### 3.1 ATOM and case formulation as abductive enterprise

In clinical psychology, abductive reasoning has been recently proposed, theorizing the possibility of a continuous interaction between data and hypotheses (Haig, 2005; Vertue and Haig, 2008; Ward et al., 1999), defined “abductive theory of method” (ATOM), and useful in order to continuously gather the “new facts”, in Peircean sense, which would remain unobserved using the standard inductive/deductive reasoning: first detecting clinical phenomena, then postulating psychological mechanisms, and finally developing and evaluating a case formulation (Ward et al., 2016). Peirce's original formulation constitutes the semiotic-logical foundation of abductive inference; ATOM can be conceived as its operationalization into a clinical and practical decision-making framework, specifically designed to manage the incomplete and defeasible evidence characteristic of psychological practice. The relationship is one of theoretical genealogy: ATOM inherits the abductive logic from Peirce and renders it actionable in applied reasoning contexts, including forensic assessment (Walton, 2004). The concept of “case formulation”, a sort of general description of the case psychological characteristic, encompassing the past history of the individual, their present condition and prognosis, and the proposed interventions, is largely based on abductive reasoning (Eells, 2007; Eells and Lombart, 2011; Sturmey, 2009) and has been considered particularly suitable to forensic psychology (cf. Sturmey and McMurran, 2011, edited collection, 2011). In fact, the process of “case formulation” takes us back to the object of diagnosis—the case—and to the application of successive steps of reasoning.

An important contribution by Hart et al. (2011) conceives formulation as the process or product of gathering and integrating diverse clinical information to develop a concise account of the relevant variables affecting a person's psychological condition to guide decision-making, applying the concept to forensic evaluation of offenders. In their conception, formulation is neither a descriptive classification nor an actuarial summarization but a theoretically informed, evidence-grounded explanation that guides intervention, risk assessment, and risk management, relying on abductive reasoning—given the epistemic constraints inherent in mental health and forensic practice. The authors emphasize that mental health disciplines lack deterministic laws that would enable deductive prediction of individual behavior: since human conduct is multiply determined, historically contingent, and sensitive to context, forensic clinicians are compelled to engage in abduction, that is, the generation of the most plausible explanation capable of rendering surprising or clinically significant facts intelligible.

In Hart et al.'s (2011) account (2011), abduction proceeds by identifying noteworthy observations, constructing a hypothesis that would make those observations expected if it were true, and adopting that hypothesis as a provisional explanatory model. The abductive hypothesis is anchored both in accepted theoretical frameworks and in the factual matrix of the individual case. Formulation, conceived in this way, becomes a disciplined mode of hypothesis construction under uncertainty rather than an impressionistic narrative or intuitive reconstruction, and functions as a bridge between nomothetic knowledge and idiographic understanding, built in the shape of a narrative structure—as explained above, not in the literary sense, but in the cognitive and epistemological sense advanced by several authors such as (Bruner 1991) and (Polkinghorne 1988). The narrative is both anchored to theoretical principles that identify what counts as causally or functionally relevant, and to empirical observations that constrain permissible explanations (Hart and Logan, 2011; Wagenaar et al., 1993). This set-up allows for a better connection between clinical and forensic diagnoses, in which the various phases of the process must be made explicit and connected to one another. Through this structure, formulation integrates historical antecedents, precipitating events, maintaining mechanisms, and contextual variables into a diachronic explanation that links past functioning to present difficulties and to future behavioral possibilities. In this diachronic architecture the future cannot be deduced, but plausible future trajectories can be inferred based on explanatory hypotheses about the mechanisms driving behavior.

Hart et al. (2011), consequently, argue that formulation, as an abductive hypothesis, must satisfy several epistemic criteria: coherence with accepted theory, internal consistency, adequate factual grounding, explanatory breadth, parsimony, and testability. According to them, testability is particularly important, because a formulation that does not generate empirically examinable predictions lacks scientific value, and does not satisfy Daubert criteria. Abductive hypotheses are therefore designed to be evaluated and revised in light of ongoing clinical data, including treatment response, behavioral change, and emergent risk indicators. This iterative cycle of prediction, observation, and revision situates case formulation within a scientific model of reasoning rather than a purely clinical or interpretive one.

Overall, Hart et al. articulate an understanding of forensic case formulation as an abductive, explanatory, and action-oriented practice. It occupies a unique epistemic space: more rigorous than intuitive clinical judgment, yet more flexible and individualized than actuarial prediction. Its purpose is not merely to describe but to explain; not merely to categorize but to generate testable, clinically actionable hypotheses; not merely to interpret the past, but to anticipate and influence the future.

In sum, clinical case formulation emerges as a rigorous yet inevitably narrative form of abductive reasoning. The clinician is a narrator who weaves signs and rules—more or less codified—into a coherent representation of psychic functioning. The quality of diagnosis depends precisely on maintaining this equilibrium between nomothetic anchors and idiographic synthesis: it can never become purely deductive, but it can—and must—remain transparent, empirically responsive, and scientifically defensible.

### 3.2 Dual-process reasoning and cognitive bias in forensic assessment

The cognitive psychological correlate of the Peircean distinction between preliminary abductive conjecture and its subsequent deductive–inductive verification is found in dual-process models of reasoning. Type 1 processing (fast, automatic, heuristic) corresponds to the spontaneous abductive leap; Type 2 processing (slow, analytic, deliberate) corresponds to the disciplined verification cycle. This correspondence, far from being merely analogical, has important methodological implications for the forensic context, as the following literature makes clear.

Raharjanti et al. (2021) have thoroughly addressed this issue, examining forensic psychiatric analysis through the lens of cognitive science, and, without invoking the concept of abduction, have made comparable suggestions. According to them, “dual-process thinking” (Kahneman, 2011) is essential for improving accuracy, transparency, and objectivity in forensic evaluations, which require a higher level of precision than common clinical ones. The authors propose that forensic psychological evaluation is, in essence, a specialized form of clinical reasoning, but one with higher stakes. Type 1 processing is fast, intuitive, automatic, associative, and based on pattern recognition and heuristics; Type 2, on the other hand, is slow, analytic, effortful, deliberate, and rule-based, requiring working memory. The authors suggest that forensic diagnoses demand a stronger and more disciplined use of Type 2 reasoning, which critically reviews and tests initial impressions. Type 2 thinking allows slowing down the process, examining evidence more comprehensively, and ensuring that the reasoning chain is explicit—something essential for forensic reports, which must be transparent, retraceable, and defensible in courts. Moreover, Type 2 reasoning—slow and analytic—should function as an internal supervisor of Type 1 (abductive and synthetic). According to the authors, examiners should explicitly adopt what they still call a hypothetico-deductive model—but which is actually intrinsically abductive—explaining how clinicians generate determinate hypotheses about mental states and capacities, deduce their implications according to legal standards, and test them through interviews, record reviews, collateral data, and mental status examination. Moreover, such evaluation should profit from what the authors call “illness script theory”, the internal theorizing about illnesses (or, for example, in civil justice, definitions of good parenting) derived from study and practice, which provides structured knowledge (“scripts”) about disorders and personality traits and their (abductive) applications to legal and forensic fields: clinical scripts and forensic scripts.

But such a process can sometimes fail, due to cognitive biases arising from the malfunctioning of the dual-process system, in which the most hurried abductions prevail and severe biases develop: anchoring bias (early impressions drive the entire evaluation), confirmation bias (selective search for supporting evidence), availability bias (overreliance on recent or vivid cases), premature closure (stopping the assessment process too early), and overconfidence (exaggerated trust in one's judgment). Such biases emerge when Type 1 reasoning is unchallenged or when Type 2 monitoring is absent or impaired. Situational stressors (tight deadlines, adversarial contexts, fatigue, and incomplete records) also increase reliance on intuitive shortcuts; and, finally, emotional reactions toward the examinee—described as forensic countertransference (Barros et al., 2014; Sattar et al., 2004)—also influence Type 1 processes and distort judgment.

### 3.3 The fractal structure of abductive inference

The integration of the idiographic and the nomothetic thus allows diagnosis to be grounded in requirements that are shared and accepted by the scientific community, according to a general principle of necessary connection between the narrative/abductive element and the inductive-deductive element. The aim of the narrative component is indeed to link together, in a sensible plot that maintains logical coherence, the data derived from the nomothetic component; and to explain and integrate any contradictions between the latter and the observations made.

This process, in reasoning on a single case, could be replicated at different levels, each step being formulated as a succession of abduction/induction/deduction, in a sort of fractal perspective. Fractal self-similarity applied to the abductive process captures something that is present in Peirce but not explicitly framed in these terms: abductive reasoning is not a single “leap” but a process that replicates itself structurally at different scales. At the micro level, each individual abductive inference (the “surprising fact” → explanatory hypothesis) undergoes the deduction-induction cycle that Peirce describes. But the outcome of that cycle—the confirmation or reformulation—itself becomes a new “fact” that feeds a higher-order abduction, with the same logical structure but on a greater plane of complexity.

This fits very well with Eco's distinction between hypercodified, hypocodified, and creative abduction: it could be read as a fractal scale of increasing complexity, where “small” abductions (hypercodified, almost automatic) provide the building blocks for hypocodified ones (diagnostic alternatives), which in turn converge toward creative abduction—the new explanatory rule. There is also an interesting resonance with Magnani (2001): his distinction between selective and creative abduction could map onto the levels of the fractal—selective abduction operates at the lower levels (choosing among already available hypotheses), while creative abduction emerges at the higher levels as an emergent property of iterative accumulation. In the particular forensic context, in which the step-by-step logic has to be explicitly documented, this fractal structure could be particularly effective, showing how clinical diagnosis proceeds through micro-abductions (individual signs, symptoms, and responses) that compose into meso-abductions (clinical patterns, diagnostic clusters) up to the final diagnostic macro-abduction—and, moreover, showing where and how the process can “derail” when narrative hypocodified hypotheses replace empirical evaluation, short-circuiting the intermediate levels. Narrative distortions in diagnoses–produced by intervening individual factors and biases, involuntary or deliberate, could be read as a “collapse of the fractal”: one jumps directly from micro-observations to macro-conclusions without the intermediate verification steps, breaking the self-similarity of the process.

Three metaphoric models come to mind: the nougat one, the net one and the musical score one. In the first metaphor, abductions integrate and keep together “stronger” scientific results (obviously intrinsically singularly or generally valid) just as albumen mixed with honey forms a sweet glue holding together the “noble” part of almonds, hazelnuts, or peanuts in the nougat. But this metaphor is not completely isomorphic to the described process, because it does not account for the direction of the connections in the explanatory process, but simply states that glue (abductions) holds together the whole. The net, on the other hand, allows for an explanation of the nature and direction of the diagnostic reasoning, and represents the multiple singular connections between one element and another. Lastly, the model of the musical score, in which harmony and melody should fit—the notes for both the abductive-idiographic and nomothetic material, evidenced by different musical signs (harmony of synchronic chords), and their connections in a diachronic melody for the abductive construction of the diagnostic plot and narration, could express the music of the whole.

At this point, it is necessary to remember that interpretative errors do not belong only to Type 1 reasoning, but also to Type 2: errors connected to the use of micro-observations to generate macro-conclusions, and to the interference of biases and confounding factors.

Moreover, it should be noted that every level is open to abductions: improper ones can occur also in the interpretation of nomothetic material: Baker (2003), for example, examines the proper forensic use of multiscale psychological inventories and emphasizes that although scoring procedures are objective, interpretation is not, requiring specialized psychometric expertise. It is consequently possible, notwithstanding the presence of validity scales detecting malingering, defensiveness, self-presentation bias or inconsistent responding, that mistakes can occur, even if one has recourse to computerized services or consults experts in the field—arguing that computerized reports are overly inclusive and risk misinterpretation, while consultants must be carefully selected due to varying degrees of knowledge and reliance on psychological instruments, as well as to contemporary and up-to-date diagnostic models, among psychiatrists and psychologists, including those working in forensics (see for instance Mulay et al., 2025; Paulson et al., 2019). Moreover, the validity of each instrument must be assessed, and cultural biases must be considered. He concludes by emphasizing the need for scientifically grounded interpretation under the Daubert standards, noting that courts increasingly scrutinize or exclude improperly validated test-based testimony, and urges clinicians to use multiscale inventories with both appreciation of their strengths and clear recognition of their limitations.

## 4 Abduction and the hypothetico-deductive model in the forensic assessment process: recommendations for the assessors

In the following section we will delineate the implications of the considerations on abduction and the hypothetico-deductive model offered above to forensic assessment, suggesting a few recommendations for practitioners. As such, we will highlight, in a temporal fashion starting from the beginning of the assessment process to its conclusion, emphasizing the passages where such decision-making aspects are involved the most, potential pitfalls for the examiner, and possible solutions.

### 4.1 Pre-assessment information and forensic confirmation bias

A first, highly salient and informative moment for the assessor is the initial one. They are confronted with the referral question, generally with a variably “objective” presentation of the examinee and of their involvement in the forensic context that is under analysis. Inevitably, the examiner will form a first, highly driven by Type I processing in Kahneman's terms, impression of the examinee. Such “micro abduction” will have the potential, if not adequately acknowledged in its provisional nature by the examiner, will pose the risk of a cascade of subsequent assumptions about the defendant that might taint the whole assessment process. The representation of the examinee that the examiner will form in this step will depend on various factors both “endogenous”, such as personal beliefs and motives, and both “exogenous”, like situational and emotional context in which the assessment takes place (Dror and Cole, 2010; Kukucka et al., 2017). This phenomenon, and the associated perils for the integrity of the assessment, is so renowned in forensic science that Kassin et al. (2013) coined for it the term forensic confirmation bias. The issue goes well beyond forensic psychological assessment, and its ethical implications have been discussed also by organisms such as the National Academy of Sciences (2009) and the National Commission on Forensic Science (2015) in the North American system. However, despite the awareness in the scientific community about the problematic outcomes of “out of control” perceptions that may affect prospective examiner's judgments, and although experimental psychology has been a privileged arena for the study of confirmation biases (e.g., Nickerson, 1998) it is common practice for forensic assessors in the legal system to receive a good amount of information regarding the examinee in advance. An eminent, yet unsettling, example of how uncritically accepting contextual information can bias reasoning on complex matters such as the one of child custody, comes from Munro's (1995) review of John Aukland Report. According to the author, social workers involved in parental fitness evaluations accepted without reserve deceitful information from the husband who made a favorable impression on them but disbelieved the wife who presented herself less favorably. Munro continues with highly embraceable recommendations for professionals working in such situations to make their first impressions explicit and consider their power in altering their reception of subsequent new information about the individuals involved, with the need of using deliberate effort to think of evidence for the opposing view.

Obviously, information such as the examinee's history, previous behavior, and their supposed or ascertained misconduct will form part of the material on which the evaluation will be run. It is important to note, however, that for certain types of forensic evaluations, prior record review is not merely permissible but epistemically necessary: in sanity evaluations and violence risk assessments, the examinee's self-report acquires interpretive meaning only against the backdrop of collateral documentation, and in cases where examinees refuse to participate, records may constitute the primary evidentiary basis. In areas such as sanity and violence risk evaluations in particular, prior record review becomes epistemically necessary to put in perspective the examinee's self-report in the light of collateral documentation. For instance, in a NGRI evaluation^1^ of an individual prosecuted for killing his partner, key “factual” elements coming from police investigations and prior records of his behavior will be key to reach a diagnostic definition. Such aspects may include the level of mental lucidity displayed by the defendant in his daily life up to the alleged criminal event, evidence of deliberate planning of the crime, as well as previous symptomatology coherent with diminished mental capacity.

Importantly, the abductive epistemology we propose does not counsel against record review, it rather advises reflective awareness of the confirmatory pull that prior material can exert. Although an effort can be made to avoid exposure to unnecessary contextual information, “blind” evaluations are actually impossible for the mental health professional performing the forensic assessment. The key is not whether one reads the records, but whether the hypotheses generated on their basis are held provisionally, remain open to revision, and are systematically tested against independently gathered data. The examiner should be as aware as possible of the potential for bias when starting the assessment, keeping in mind that the information received about the examinee has been inevitably “filtered” by its source. Even more central for an accurate assessment is the ability of the practitioner not to crystallize initial, implicit abductive reasoning as the truth, but to remain in active search of alternative explanatory possibilities, using deductive and inductive procedures to collect further information.

### 4.2 First encounter, thin slices, and self-presentation strategies

The actual first encounter between the examiner and the examinee is yet another turning point where heuristical reasoning, if happening inadvertently, can have a cascade effect on the assessment process that will follow. Such “first impressions”, also called “thin slices” or “zero acquaintance judgments” (see Wood, 2014 for an extensive review) are fundamental in virtually every human interaction, and even more so in rater-based assessments, in and outside the forensic world. Such rapid, unconscious decisions–that again, could be seen as “micro abductions”–are categories that people use as an aid to perceive, categorize and integrate information coming from the external world, and are particularly at work when the rater deals with another individual's personality and behavior (Feldman, 1981; Gingerich et al., 2011). As research shows (Wood, 2014), such impressions are not inherently wrong and can possess varying levels of accuracy also depending on the object of the rating and on the intentions of the examinee to self-present in a particular way. “Malingering”, the deliberate production of false or grossly exaggerated physical or psychological difficulties to obtain secondary advantages (e.g., eluding criminal prosecution) is the most prominent example of self-presentation strategies that might bias the evaluation. Other factors that may affect the accuracy of the rater's appraisal of the examinee include more “basic”, facial characteristics of the latter, and judgments based on such features show reliability quite close to chance (Porter et al., 2008). For instance, when judging trustworthiness and perceived honesty, assessors will rely also on facial features such as “babyfacedness”, symmetry, “softness”, and attractiveness (Bull, 2006; Zebrowitz et al., 1996), and such automatic processing is also neurophysiologically rooted (Rule et al., 2013; Todorov, 2008). Finally, it seems that personality characteristics vary on the average easiness by which they are detected in first impressions contexts: extraversion, for instance, results to be easier to judge than other traits (Borkenau and Liebler, 1992; Todorov et al., 2015).

As anticipated, although such automatic, “micro-abductive” attributions may be useful shortcuts in everyday life situations (e.g., Feldman, 1981) inadvertently let them affect the following observations can lead to errors in judgment and the effects on rater-based assessments in particular can be dramatic, especially with high-stake decisions like those taking place in the forensic and legal process. Examining such considerations in relation to a few cases of documented wrongful convictions deliberated by a court in Canada, and on the criterion of “beyond a reasonable doubt” to ascertain guilt and innocence in courts, Porter and ten Brinke (2009); Porter et al. (2010) developed their Dangerous Decisions Theory (DDT). In such a framework, the authors illustrate how attributions based on a defendant's face and emotional expressions play a major role in initiating a series of “dangerous” decisions concerning their credibility wrongful convictions. In an experimental laboratory setting, they provided participants with two vignettes describing criminal acts of varying severity, each paired with a photo of the defendant previously rated as highly trustworthy or untrustworthy. They then asked the participants to assess the guilt or innocence, and ratings of confidence were reported after the presentation of each piece of increasingly incriminating and exonerating evidence. As predicted by DDT, participants required less evidence to arrive at a guilty verdict of (the same) crime compared to a perceived trustworthy person, particularly in cases of severe crimes (murder). Although in a simulated jury study context, the study findings suggest that crime severity might increase motivation and emphasize further initial impressions, in a sort of “tunnel effect”. In such more high-stake decisions, participants were also more confident in their verdict, reminding once again how context can shape the assessor's mental set-up in decision making.

If the risk of bias present in (unacknowledged) first impressions is particularly relevant for common law legal systems because of the role played by jurors in the verdict formulation, such mechanisms can well be at play also in similar forms of trial jury in the Italian legal system such as Corte d'Assise. And even more centrally to the issue of forensic case formulation, the caveat of not relying on “unacknowledged” first impressions leads to specific methodological recommendations for the psychologist/psychiatrist carrying out the evaluation.

### 4.3 Multimethod and Process-Focused assessment as epistemological safeguards

In this regard, above all is a wisely planned collection of information on the examinee by means of at least two methods of measurement (e.g., self/informant report, implicit, structured rating, and physiological) and their subsequent careful integration with other information available (e.g., referral question, anamnestic information, behavioral observations, external records, collateral informant reports, and consultation). In this context, psychological tests can be conceived as the quantitative, yet idiographically relevant, base of the evaluation, and collateral, external information serve as the overarching frame to put the findings in a meaningful context. Such wise yet rigorous use of multimethod assessment represents the primary and most powerful tool psychological assessors have in and outside the forensic context to overcome the perils coming from external and internal influences during case formulation. In their seminal review of data from more than 125 meta-analyses on test validity and 800 samples examining multimethod assessment, Meyer et al. (2001) favorably concluded on the robust and compelling validity of psychological tests, which is comparable to the one of medical tests, stressed the uniqueness of the information provided by distinct forms of them, and, importantly, highlighted that clinicians who rely exclusively on interviews are prone to incomplete understandings.

The essential need of multimethod assessment in forensic practice is clear from the above discussed specificities of clinical reasoning applied to the legal context, and can be briefly summarized in the increased risk of specific distortions of both the assessors' evaluation (e.g., the “tunnel vision” discussed above) and on the examinees' self-presentation manipulations. Yet, it has been only in the last decades that forensic psychological assessment has started to systematically include structured assessment tools (i.e., psychological tests) to integrate an approach almost exclusively based on “plain” clinical judgment (Neal and Grisso, 2014). Notwithstanding forerunner remarks from (Borum et al. 1993) back in 1993, skepticism about test-based mental assessment has often been raised by different scholars in favor of “unassisted” clinical reasoning (e.g., Litwack, 2001; Montgomery, 2005). Moreover, even when implying formal psychological assessment, a selective emphasis on monomethod evaluations, and on self-reports in particular, has been documented starting from surveys on the curriculum of doctoral clinical training programs in the United States (Ready and Veague, 2014) and has been echoed in the research literature as well (e.g., Bornstein, 2007, 2003). And as Robert Bornstein (2007, p. 3) wittily put it: “Given people's limited self-awareness and inclination to dissimulate (i.e., to “fake good” or “fake bad”), psychology's reliance on self-report tests is a recipe for failure in the laboratory, clinic, and courtroom”.

Actually, an “actuarian vs. intuitive clinical” approach debate does not seem of utility, as it would not be useful to select idiographic vs. nomothetic reasoning. Rather, a clinically sound, yet rigorous multimethod assessment can aid in avoiding bias and simultaneously add individualized richness and increase the predictive value of the evaluation (Hopwood and Bornstein, 2014). This is becoming more significant in a legal system that is increasingly focused on rehabilitation, such as the probation process inherently demonstrates. The discourse has shifted, as well, from unstructured clinical judgment vs. actuarial prediction toward a more nuanced contrast between structured professional judgment (SPJ) and actuarial approaches (cf. Neal and Grisso, 2014). The abductive framework we propose is compatible with and supportive of this shift: it provides the epistemological rationale for why SPJ represents a methodological advance, insofar as it preserves the inferential flexibility that abductive reasoning requires while maintaining the empirical constraints that nomothetic data supply.

Process-Focused (PF) model of multimethod assessment is a validated paradigm to guide psychological evaluations that seems particularly relevant in terms of aiding the clinician overcoming the shortcomings of narrow-sided, excessively abductive inferences, while at the same time maintaining the richness of the clinical case formulation. Conjugating the benefits of multimethod assessment and idiographic vision, PF model can complement the traditional, nomothetic approach. PF assessment can aid the examiner selecting instruments to use, and develop person-centered predictions that are very much needed when dealing with key issues of legal assessment, such as competence to stand trial, culpability, risk towards oneself and others, and parental fitness. PF was initially developed to explain the apparently “ostensibly conflicting results” that can be gained from different sources of assessment (e.g., self-report as the MMPI and performance-based personality tests such as the Rorschach Test), which tend to very lowly correlate, typically with a r from 0.20 to 0.30 (Meyer et al., 2001). Bornstein wisely developed such bottom-up method drawing from a variegated corpus of research spanning from (McClelland et al. 1989) work on implicit and explicit motives, to (Whitely 1983) studies on construct validity that combines traditional psychometric and information-processing approaches, as well as investigations on the effects on test score variability resulting from experimentally manipulated instructional sets (e.g., Steele and Aronson, 1995), examinee's emotional states (e.g., Hänze and Meyer, 1993), and examiner's characteristics (e.g., Butcher, 2002).

In the PF framework, the apparently unexpected discrepancies between different measures of the same construct are actually understandable in the light of the distinct psychological processes activated by test takers while they respond to the tasks of different methods of measurement (Bornstein, 2002). To make a straightforward example, when responding to a self-attribution test–in other words, a self-report– or to a stimulus-attribution test–such as the Rorschach Inkblot Method– the basic psychological systems of emotion, cognition, motivation, and social behavior will be differently in action. The tasks requested the examinee to complete will be, in fact, different and so will be the meaning of test scores. In the first case they will reflect the degree to which the person attributes a set of traits, feelings, thoughts, motives, behaviors, attitudes, or experiences to themselves. In the latter, test scores will reflect the meaning attributed by the individual to a variably ambiguous stimulus, with both stimulus characteristics and the person's cognitive style, motives, emotions, and need states contributing to the process. What is key in reconciling the observed discrepancies is the convergence in predicting behavior (Bornstein, 2002). In quite a typical scenario from the legal context, parental fitness evaluation for instance, when assessing an individual's personality, implicit measures, such as the Rorschach, might signal problems in impulse control, whereas self-attribution test scores might be silent for this aspect. Such discrepancy might suggest the examinee's attempt to deny impulse dyscontrol or lack awareness of it. In a different scenario from a “not guilty by reason of insanity” evaluation, it would be noteworthy, and a potential sign of a manipulated self-presentation, to find the inverse pattern: high scores on self-reported problems with impulse management and indicators of more controlled, rational behavior on implicit personality methods. In both instances, a recursive re-consideration of test findings considering collateral information will be helpful to generate and confirm the best interpretative hypothesis. Notwithstanding the encouraging evidence of the PF model's utility for operationalizing and empirically assessing test score validity (Bornstein, 2011; Bornstein et al., 1996, Bornstein et al., 1994), its application to forensic case formulation is quite a novel development of assessment science and practice, and further investigations on the matter will be particularly valuable. A recent, interesting example is the application of PF multimethod assessment for assessing risk in forensic populations (Natoli et al., 2023).

As will be clear to the reader from the arguments offered so far, the benefits of structured approaches to case formulation, are multiple and known in the specialized literature across legal, medical and mental health fields (e.g., Ægisdóttir et al., 2006; Guy, 2008; Haynes et al., 2011). What is noteworthy and of added-value in the multimethod assessment framework applied to the forensic context is the methodological and practical implications to assist the assessor in setting-up the process and support decision-making tasks.

A first implication of endorsing the multimethod assessment framework in forensic practice, and/or its PF extension, is the most obvious yet powerful one: rely on structured assessment (i.e., psychological tests) to integrate information coming from external sources and interpretations based on clinical judgment with diverse types of measurement. Beyond augmented validity of the evaluation, in forensic practice this has the fundamental advantages of (a) minimizing the impact of micro-abductions generated in the examiner by contextual information and first encounter with the examinee; (b) allowing for more control on potential distortions in the individual's response to single measures. Regarding the latter, the aforementioned deliberate, intentional production of symptoms for personal advantage (e.g., malingering and fake bad) or, to the contrary, the attempt to deny undesired self-attributions (e.g., “fake good” or social desirable responding), both processes that are typical of the forensic setting, can be more systematically controlled for using multiple tests. This can be done through *ad hoc* measures (i.e., the Inventory of Problems: IOP, Viglione et al., 2017), by multidimensional instruments with validity scales (e.g., the MMPI; Butcher et al., 2001), or with a sophisticated integration of physiological measures as outlined, for instance, in PF multimethod assessment for risk prediction (Natoli et al., 2023).

Furthermore, the validity of monomethod assessments can be limited, in and outside the forensic context, by the intrinsic differences that will emerge on the same object–be that a psychological trait, a behavior, an event–if it is recorded from different perspectives. The literature shows that accounts of the same experience can vary significantly between individuals, other informants (such as parents), and clinical chart descriptions (Hardt and Rutter, 2004; Henry et al., 1994; Widom and Morris, 1997; Widom and Shepard, 1996). To note, similarly to the low average correlations yielded by self-attribution and stimulus-attribution tests discussed above, systematic divergencies can arise also from structured assessments conducted on the same objects, especially for complex constructs such as personality and personality disorders (Gritti et al., 2016). This is particularly true for less observable and less frequent personality disorders and invites once more not to “trust” a single method of measurement. Many confounds can intervene, for instance, in the examinee's capacity to accurately describe their emotions, behaviors, thoughts, and motivations (Ganellen, 2007). Self-perception, largely depends on implicit memory systems that cannot be straightforwardly put into words (Berlin, 2011; Roediger, 1990) and such an expression will depend also on the individual's level of insight, cognitive ability to understand item content, and affective variability (Huprich et al., 2011). In this vein, the construct of narcissism stands as emblematic in forensic assessment, for its role as a risk factor of violence and low treatment responsiveness in forensic populations according to a recent systematic review (Keune et al., 2022) and the possible difficulties in insight capacities of individuals with narcissistic functioning (e.g., Handler and Hilsenroth, 2006; Miller et al., 2010). As such, multimethod assessment of narcissism and grandiosity have proved particularly fruitful in capturing the complexity of such a condition and some of its socially problematic behavioral correlates such as hostility (Gritti et al., 2018, 2021).

### 4.4 Critical issues and guidelines for forensic multimethod assessment

When employing a multimethod assessment framework, it is essential to carefully consider both the selection of assessment tools and the sequence in which they are administered. Although a full discussion of these methodological aspects falls outside the aims of the present work, some recommendations can be offered to minimize bias and support examiner decision-making. Firstly, instruments should be selected considering the specific goals of the evaluation and anchoring to the scientific literature and classification by the process activated in the examinee (see table 1 from Natoli et al., 2023). For instance, when dealing with defendants that are suspect of malingering, but their reality test and personality functioning are to be examined as well, a combination of methods to control for distorted self-presentations, and both implicit (e.g., the Rorschach) and explicit (e.g., the PAI) methods will be likely useful. Despite the relatively scarce literature on the topic, specific attention should be given also to the order in which tests are administered. In the context of disability claimants' evaluations, for instance, (Guilmette et al. 1996) empirically showed that test order administration can affect the results of the examination, with this being an example of “non-test factor”.

A second warning for the multimethod oriented assessor should be the apparently “naïve”, yet not so improbable in light of the risk of bias coming from first impression and contextual information, is the one of avoiding the “cross-use” of information from a test while interpreting the other. This is particularly relevant when operating with tests leaving more latitude for the examiner's judgment (e.g., performance-based tests) and extends also to the risk of contaminating the interpretation of test findings with anamnestic information collected with the examinee. In this regard, more structured approaches to history-taking, such as the one originally proposed by Karl (Menninger 1952) drawing from the procedures used at the Boston Psychopathic Hospital and with subsequent developments at the Menninger Clinic, can both have the advantage of being comprehensive and avoiding a potentially biased, or impressionistic, focus on too narrow areas of functioning.

Finally, it might be useful to remember, in light with (Bonfantini and Proni 1983), also the apparently “logically strongest” conclusion might obscure its abductive and potentially fallacious nature. The final step of assessment will therefore be for the assessor to travel through the decision-making process again and critically reconsider the potential “shortcuts” taken, choose the most empirically grounded diagnostic explanation and don't forget its intrinsic “probabilistic” nature.

## 5 Case examples of potential pitfalls in forensic case formulation

When experts fail in following recommendations similar to what has been exposed above, mistaken evaluations can follow, directly connected either to the erroneous or manufactured coordination of clinical observations and test-based assessment, or to the total disregard of some of the various sources which should be considered and pondered together. In such cases, the harmony of the parts of the formulation vanishes, and the conclusions do not logically fit with the observations and nomothetic data.

Coming to the details, the most frequent errors that can be observed in practice refer to different parts of the diagnostic procedure: the erroneous use of diagnostic abductions (Type 1 reasoning), the interpretation of the nomothetic material, and the construction of the “nougat”, or “net”, or “melody” of connected data. As we have mentioned, in such cases what should appear as a harmonious abductive weaving integrating “almonds” or “knots” or “chords” in a complex and signifying pattern, appears torn: data are omitted, cohesion lacks, observations and results are incorrectly reported or maliciously altered. In such cases, if a harmony is present, it is produced through a forcing of details, as if, in composing a jigsaw puzzle, some pieces were pressed against one another and deformed in order to produce a joint, or cut and reshaped, or even thrown away if they do not fit with the desired preconceived image.

Here are didactic exemplificatory cases from forensic practice: the first refers to the partial use of the clinical abductive observations, with some important material being not mentioned (omission in recognizing the fractal), and the result is a mistaken final formulation:

In a criminal case of negligent injury in a Southern Italy court, a Medical Doctor (“Dr. Bianchi”) received an accidental overdose of a contrast agent during a routine diagnostic procedure. Court-appointed experts diagnosed him with PTSD, relying primarily on clinical certificates and interviews. However, the defense party consultant identified a constellation of red flags pointing toward symptom fabrication or exaggeration. Both self-report tests administered yielded valid results (no more than 2 items of the V scale with a “True” response, raw score of X scale < 34 or > 178, no more than 12 invalid/blank items, and all PDs scales base rates < 59 for the MCMI-III; ICN < 64, INF < 60, PIM < 57, and NIM < 70 for the PAI). Of note, the MCMI-III scores suggested a tendency toward a contradictory self-presentation, with a BR score of 80 on the Y scale—indicating a favorable self-presentation—alongside a concurrent tendency to portray oneself in self-deprecating terms (*Z* = 67). In terms of clinical scales interpretation, the psychodiagnostic tests told a conflicting story as well: the Millon (MCMI-III) revealed a pre-existing dysthymic profile with anxious and obsessive features within a personality structure oriented toward minimizing pathology—yet depressive symptoms broke through regardless, producing internally contradictory data. On the other side the PAI, while flagging PTSD-related items, simultaneously returned stress levels within the normal range—a finding fundamentally incompatible with an active post-traumatic condition and suggestive of symptom amplification. Moreover, the complainant held a postgraduate diploma in Forensic Psychiatry, making him an insider thoroughly familiar with diagnostic criteria, assessment procedures, and the symptom profile of PTSD; and when the defense party consultant directly asked during the evaluation whether he had experience in diagnosing PTSD, denied any such expertise—a claim flatly contradicted by his own professional advertising, where PTSD was listed among his core specializations. Finally, the PTSD diagnosis itself rested on shaky nosological ground: since criterion A requires exposure to actual or threatened death or serious injury, yet no documented evidence in the international literature linked the substance in question to life-threatening outcomes at the dosage administered, and a prior court-ordered medical evaluation had already excluded a causal connection between the incident and the somatic symptoms the complainant attributed to it.

In other cases, experts deliberately do not mention a few data that emerged in the observation, even denying some important aspects of reality which could have cast a bad light on their formulation (rupture of the fractal). Here is another example:

In a custody dispute in a town of northeastern Italy, the party-appointed expert (CTP) for the father, Mr. “Netto” systematically distorted the psychological assessment by omitting critical test findings—most notably the father's elevated vulnerability index for affective disorders emerged from the Rorschach Test (R-PAS method)—while inflating minor scoring anomalies in the mother's Rorschach report to construct a narrative of her cognitive impairment. To note, the evaluation was limited in that it relied on a single testing instrument (i.e., the Rorschach Test). The CTP also built factual counter-narratives unsupported by evidence, such as claiming the mother had been the aggressor in documented domestic violence episodes, and attempted to discredit her through trivial and unrelated matters (unpaid fines, the use of a relative's bank card). Meanwhile, Mr. Netto engaged in pervasive controlling behaviors during the evaluation itself—showing up uninvited at public events in which the son was present, conducting nighttime surveillance near the mother's home, and erupting in rage before social workers. The court-appointed expert (CTU), however, saw through these maneuvers: she discarded the inappropriate abductions of the father's CTP, documented his pattern of rationalization and denial, identified his complete inability to mentalize the child's emotional states, and noted that the 8-year-old boy was showing clear signs of developmental trauma—emotional constriction, avoidance of expressive play, and normalization of violence. The CTU concluded by recommending controlled contact with the father, intervention of the social services, collocation in the mother's home and urgent therapeutic intervention for the child, recognizing the mother's consistent cooperation and genuine attunement to her son's needs throughout the process.

Still other experts arrive at altering the results of nomothetic material, interpreting it in a personal and deceitful way, to harmonize it with a mistaken set of abductions (manipulation and faking of the fractal). Here we report a case from many years ago:

In another custody dispute in a small town in Liguria, the court-appointed expert (CTU) equalized two profoundly unequal parents by attributing to the mother, Mrs. “Rossi”, fabricated psychopathological constructs: “reality slippages”, “pathological enmeshment”, and a “false Self” in her 11-year-old son “Mattia”, none confirmed by the standardized assessments he herself had ordered. Meanwhile, he downplayed the father, Mr. “Bianchi”s, diagnosable Narcissistic Personality Disorder with grandiose-malignant features at SWAP-200, noting only, with remarkable understatement, that he displayed “occasional irony”. Moreover, he selectively omitted passages from his own consultant's report documenting the father's hostility, contempt, and manipulativeness—the same father who during the evaluation period sent the mother 147 emails in a single weekend and showed up uninvited at Mattia's school chess tournament wearing a t-shirt reading “World's Best Dad”. The party-appointed expert demonstrated that the child's psychodiagnostic profile showed post-traumatic markers rather than the psychotic-level defenses observed by the CTU, and that Mattia's outstanding scholastic and social functioning flatly excluded pathological fragmentation. Most gravely, when Mattia disclosed new details concerning further memories of father's violence, the CTU misreported his words and proposed the boy's removal to a residential facility.

Surely, it is not easy to arrive at clear formulations in many very complex and debated situations found in forensic practice: as can be seen, two of our forensic clinical vignettes reported above refer to the refusal of a parent by a child, and the connected construct of parental alienation. It is in this field that an abductive procedure can be very useful, as Garber (2020) asserts in a seminal paper, to minimize confirmatory bias—the tendency to interpret information in ways that confirm pre-existing beliefs while disregarding contradictory evidence. Drawing on extensive literature, Garber warns that clinicians who begin with a suspicion of either alienation or estrangement often tend to confirm that initial hypothesis, violating ethical guidelines that require evaluators to minimize bias, employ multiple methods and explore competing hypotheses, all through a transparent reasoning. The paper's central contribution is a systematic review of the resist/refuse dynamics literature, building from Kelly and Johnston's (2001) seminal reformulation of parental alienation syndrome: through a qualitative meta-conceptualization of nine key publications, Garber identifies 13 non-mutually exclusive variables that evaluators must consider in addressing such complex issue: the child's temperament; the objective quality of relationships with both parents; suggestibility; developmentally appropriate affinity; acute reactivity to specific events; event- or time-dependency; resistance to separation from the preferred parent across contexts (enmeshment); generalized separation anxiety; avoidance of parental conflict at transitions; avoidance of stimuli incidental to the refused parent's environment; “culture shock” from disparate parenting styles; the refused parent's sensitivity as caregiver (estrangement); and the preferred parent's undermining of the child's relationship with the other (alienation). Garber emphasizes that these factors are mutually compatible—any case may involve multiple factors simultaneously in varying degrees—representing an advance beyond the single factors models.

Garber's paper represents an example of admonition to reasoning accuracy. More generally, as Raharjanti et al. (2021) explain, several strategies are possible, nearly all aimed at improving Type 2 monitoring over Type 1 intuitions, and to clearly point out the diagnostic procedures. Since the training period, it would be necessary to explicit teaching of clinical reasoning and cognitive bias, giving serial-cue feedback aimed at exposing reasoning steps and not only final conclusions of diagnostic formulations, and providing trainees' exposure to varied forensic cases to expand script repertoires; forcing cognitive strategies to interrupt automatic reasoning and encouraging uncertainty, reflection and alternative hypotheses, and, finally, creating supportive, low-pressure environments to reduce Type 1-dominant shortcuts.

This is the essence of differential psychodiagnosis: from each potential abductive “rule”, one infers additional signs or findings that should be present if the rule is correct, and one returns to the patient's record, the mental status examination, or additional tests to check whether these consequences actually appear. Clinical reasoning thus becomes a sequence of abductive refinements, in which hypotheses are progressively constrained and clarified, until a complex and exhaustive evaluation can be produced.

## 6 Ethical considerations and conclusions

The result of our analysis, in conclusion, suggest that an informed forensic clinician should be very careful in his analytical evaluation of the results, considering all the diagnostic information he has gathered without omissions, connecting them in a meaningful way and explicitly documenting every step of their logical path, in which every abductive rule should constitute the basis of a following one. Training academies and institutes in forensic psychology should encourage such attitude in trainees, as Raharjanti et al. (2021) note, and should adopt teaching politics aimed at reducing confirmation bias, foster a mental attitude of systematic doubt and reflection about the processes, and should explicitly warn about the pitfalls and perils of the formulation task.

From the present paper, however, and besides the suggestions to improve the scientific level of forensic diagnostic formulations, it might be possible to draw the first lines of criteria for the evaluation of the forensic psychological expert from a deontological and ethical perspective. Ethical and deontological codes, in fact, contain recommendations that could be substantiated by an approach like ours. This is crucial for enhancing epistemic transparency throughout the documentation of inferential reasoning.

The American Psychological Association (2017) Ethical Code (2017) establishes that professional judgments must rest on the established scientific and professional knowledge base of the discipline (Standard 2.04), and that opinions expressed in reports, recommendations, or forensic testimony must be grounded in information and techniques sufficient to substantiate the findings (Standard 9.01a). Consistently, the APA Specialty Guidelines for American Psychological Association (2013, Guideline 9.02) further emphasize the importance of relying on multiple sources of information, recommending that forensic practitioners avoid depending on a single data source and corroborate key data whenever feasible. When uncorroborated data are used, practitioners are expected to acknowledge their status, along with the associated strengths, limitations, and rationale for their inclusion.

Similarly, such specialty guidelines stress both the importance of anchoring the selection of testing measures to the scientific literature (EPPCC Standard 9.02; AERA, APA, and NCME, in press) and the necessity to consider the examinee language preference and ability to understand the proposed material (EPPCC Standard 9.02). Importantly in terms of increased awareness and communicability of the steps taken by the assessor in interpretation of the assessment findings, such standards recommend making explicit the potential strengths and limitations in their methodology. This is particularly relevant when working, for instance, with individuals from under-represented populations, for which specific norms are not available and adaptations are necessary.

The importance of being sensitive towards cultural issues, especially when dealing with examinees from diverse background and belonging to minorities, but also of being mindful regarding potential personal biases of the examinee, is similarly emphasized in the APA Specialty Guidelines for Forensic Psychology (2013). Forensic practitioners are expected to recognize how their own cultural background, beliefs, values, and potential biases may compromise their competence and impartiality, and to take appropriate corrective measures when such factors arise (Guideline 2.07). Equally, they are called upon to appreciate individual and group differences—including those related to age, gender, ethnicity, language, disability, socioeconomic status, and other relevant factors—and to ensure they possess the necessary expertise to work competently with diverse populations, seeking additional training or consultation when needed (Guideline 2.08).

The Italian Code of Ethics (Consiglio Nazionale dell'Ordine degli Psicologi, 2013) for Psychology, on its part, is more diffuse, but adequately hits the target in stressing the importance of mindful decision making in assessment practice. Article 5 requires psychologists to maintain adequate and up-to-date professional competence, to employ only those theoretical and practical tools for which they have acquired sufficient expertise, and to ground their methodologies in identifiable scientific sources. Article 7 further mandates that psychologists carefully evaluate the validity and reliability of the information underpinning their conclusions, present alternative interpretative hypotheses when appropriate, acknowledge the limitations of their findings, and base their professional judgments exclusively on direct knowledge or adequate and reliable documentation.

It is therefore possible to find a substantial agreement between doctrinal and methodologically inspired and ethical recommendations: the necessity to dispose of sufficient documentation and to refer to scientific and professional knowledge; to use recognized and updated techniques and to secure transparency and visibility of the diagnostic reasoning process, considering and documenting possible alternatives.

From this point of view, a diagnostic error could also become an ethical error: far from a logic of punishment as an end in itself, in our opinion it might be very useful that the professional associations of practitioners dedicate not only pre-service, but also in-service training, to the development of competence and skills in forensic sector. Forensic practitioners' errors and misbehavior can, in fact, influence the reputation of the entire psychological profession and the perception the society has of the justice system, considering the visibility, the possible dissemination and the interest that some cases can arouse in the public.

## Data Availability

The original contributions presented in the study are included in the article/supplementary material, further inquiries can be directed to the corresponding author.

## Footnotes

1 “Capacità d'intendere e di volere” in Italian criminal law.
